# Book Reviews

**Published:** 1893-03

**Authors:** 


### *BOOK REVIEWS.*

---

Diseases of the Rectum, Anus and Sigmoid Flexure. By Joseph M. Mathews, M. D., Professor of Principles and Practice of Surgery, and Clinical Lecturer on Diseases of the Rectum, Kentucky School of Medicine; Consulting Surgeon to Louisville City Hospital; Visiting Surgeon to St. Mary and Elizabeth Hospital, etc. D. Appleton & Co., New York.

In the excellent introduction to his book, Dr. Mathews states that "diseases of the rectum have not received that careful attention of the medical profession which their importance demands. From time immemorial diseases of the rectum have been in the hands of the charlatan." The literature on rectal diseases, until recently, has been comparatively meager, and the motive of this work is to place this subject before the profession on a plan commensurate with its importance. Dr. Mathews was one of the American pioneers in this branch of surgery, and his many years of rich experience enable him to speak with authority. The work is not a compilation, but a record.

"I have written this book because of a desire to record my individual experience of fifteen years as a rectal specialist, in answer to the demand of my students and friends. During this time I have learned that many things that are taught are not true, and that many true things have not been taught. I have therefore not taken other men's opinions as my guide, but have accepted as truths only those things which could be substantiated by fact."—[*From the Preface.*]

The author has made a valuable contribution to the literature of this subject. The introductory chapter presents, in a general way, some suggestions and rules to be observed in examination, diagnosis, etc. The chapter on antiseptics and disinfection in rectal surgery is one of the most practical. The chapters on hemorrhoids are especially instructive and valuable. For internal hemorrhoids the author advocates radical methods. He states that there are *thirteen* recognized operations for internal piles, but really only two which claim much attention from surgeons, and these are the ligature and the clamp and cautery. He disapproves of carbolic acid injection, and Whitehead's operation seems to be disposed of pretty well in seven objections. The treatment by ligature is then described in detail, and the author reports one thousand operations by this method without a death or a case of septic infection. Other chapters discuss fissure, fistula, malignant and non-malignant stricture of the rectum, prolapsus ani, disease of the sigmoid flexure, etc. The book is provided with six full page chromo-lithographs and numerous illustrations. We think Dr. Mathews' book will be generously received and appreciated. L. B. G.

---

A TEXT-BOOK OF PRACTICAL THERAPEUTICS. By Hobart Amory Hare, M. D., Professor of Materia Medica and Therapeutics, in the Jefferson Medical College of Philadelphia, Physician to St. Agnes's Hospital and to the Jefferson Medical College Hospital, etc. Third edition, enlarged and revised. Lea Brothers & Co., Philadelphia.

The fact that this work has reached its third edition within two years is evidence that it is very much liked. In this edition the author has incorporated our information of the newest drugs, such as Solophen, Europhen, Phenocoll, Piperazine, etc., and also given the latest observations on such older ones as Salol, Phenacetine, Menthol, etc. A classification of drugs according to their physiological properties is also added, although the alphabetical arrangement is adhered to in the descriptive text. Dr. Hare's work is concise and practical, and excellently adapted to the needs of both student and practitioner. This is essentially a *new* book, brought down to date, free from unimportant and tedious detail, and we are sure it will obtain a well-deserved popularity.

---

THE ANATOMY OF THE PERITONEUM. By Franklin Dexter, M. D., Assistant Demonstrator of Anatomy, College of Physicians and Surgeons, New York. With twenty-eight illustrations. D. Appleton & Co., New York.

Anything which will simplify the anatomy of the peritoneum, with its various reflections, will be hailed by students with delight. This is the purpose of this small work. The author states that "there is no way of obtaining a clear idea of the peritoneum except through a knowledge of its development," and on this theory he traces the development of the alimentary canal, with its mesenteries, omenta, peritoneum and abdominal organs, through all their stages from their most primitive forms in the embryo up to the completed and complicated organism. The book is illustrated with colored diagrams which assist in making the matter clear. The author's self-imposed task is a difficult one, but he has been very successful with it, and those who wish to embellish their anatomical knowledge with the true conception of the peritoneum, will do well to read this volume.

---

MANUAL OF PRACTICAL MEDICAL AND PHYSIOLOGICAL CHEMISTRY. By Charles E. Pellew, E. M., Demonstrator of Physics and Chemistry in the College of Physicians and Surgeons (Medical Department of Columbia College), New York. Honorary Assistant in Chemistry at the School of Mines, Columbia College. With illustrations. New York, D. Appleton & Co.

The author furnishes to the medical profession in this book one of the best and most comprehensive treatises on medical and physiological chemistry, including urinary analysis, that has been published. The chemistry of digestion, secretion and excretion, analysis of drinking water, the comparative value of foods, and urinary analysis, are the principal subjects treated. The illustrations and plates are superb, and the tests are simplified and taught in the most lucid manner. The book is rather advanced for the beginner, as it deals largely with that more complex part of chemistry, the carbon compounds. It is a valuable work for the student or practitioner of medicine.

---

CHARACTERISTICS. By S. Wm. Mitchell, M. D., LL. D., Philadelphia. The Century Company, New York. Price $1.25.

Those who had the pleasure of reading this story of Dr. Mitchell's as it appeared serially in the *Century Magazine* will be glad to know that it is now accessible in book form. This is something more than a story; in it the distinguished author, under the disguise of Dr, North, has given us some interesting pages out of his own history; besides discoursing pleasantly on human nature and its impulses, with the various aspects of which his wide experience has brought him into very close contact. It is not often that one of our profession achieves fame alike in medicine and general literature, but Dr. Mitchell has deservedly accomplished this distinction. This book, while not medical, has much of interest and value for us doctors. We ought all to read it.

